# Effectiveness of nivolumab affected by prior cetuximab use and neck dissection in Japanese patients with recurrent or metastatic head and neck cancer: results from a retrospective observational study in a real-world setting

**DOI:** 10.1007/s10147-021-01900-4

**Published:** 2021-04-08

**Authors:** Shin Kariya, Yasushi Shimizu, Nobuhiro Hanai, Ryuji Yasumatsu, Tomoya Yokota, Takashi Fujii, Kiyoaki Tsukahara, Masafumi Yoshida, Kenji Hanyu, Tsutomu Ueda, Hitoshi Hirakawa, Shunji Takahashi, Takeharu Ono, Daisuke Sano, Moriyasu Yamauchi, Akihito Watanabe, Koichi Omori, Tomoko Yamazaki, Nobuya Monden, Naomi Kudo, Makoto Arai, Shuji Yonekura, Takahiro Asakage, Akinori Fujiwara, Takayuki Yamada, Akihiro Homma

**Affiliations:** 1 Department of Otolaryngology, Head and Neck Surgery, Okayama University Hospital, Okayama, Japan; 2 Department of Medical Oncology, Faculty of Medicine and Graduate School of Medicine, Hokkaido University, Sapporo, Japan; 3 Department of Head and Neck Surgery, Aichi Cancer Center Hospital, Nagoya, Japan; 4 Department of Otolaryngology, Graduate School of Medical Sciences, Kyushu University, Fukuoka, Japan; 5 Division of Gastrointestinal Oncology, Shizuoka Cancer Center, Nagaizumi, Japan; 6 Department of Head and Neck Surgery, Osaka International Cancer Institute, Osaka, Japan; 7 Department of Otorhinolaryngology, Head and Neck Surgery, Tokyo Medical University, Tokyo, Japan; 8 Department of Otorhinolaryngology and Head and Neck Surgery, The University of Tokyo Hospital, Tokyo, Japan; 9 Head and Neck Oncology Center, International University of Health and Welfare, Mita Hospital, Tokyo, Japan; 10 Department of Otorhinolaryngology, Head and Neck Surgery, Hiroshima University Hospital, Hiroshima, Japan; 11 Department of Otorhinolaryngology, Head and Neck Surgery, University of the Ryukyu Hospital, Nishihara, Japan; 12 Department of Medical Oncology, The Cancer Institute Hospital of Japanese Foundation for Cancer Research, Tokyo, Japan; 13 Department of Otolaryngology, Head and Neck Surgery, Kurume University Hospital, Kurume, Japan; 14 Department of Otolaryngology, Head and Neck Surgery, Yokohama City University Hospital, Yokohama, Japan; 15 Department of Otolaryngology, Head and Neck Surgery, Saga University Hospital, Saga, Japan; 16 Department of Otolaryngology, Head and Neck Surgery, Keiyukai Sapporo Hospital, Sapporo, Japan; 17 Department of Otolaryngology, Head and Neck Surgery, Kyoto University Hospital, Kyoto, Japan; 18 Division of Head and Neck Cancer Oncology, Miyagi Cancer Center, Sendai, Japan; 19 Department of Head and Neck Surgery, National Hospital Organization Shikoku Cancer Center, Matsuyama, Japan; 20 Department of Otorhinolaryngology, Hirosaki University Graduate School of Medicine, Hirosaki, Japan; 21 Department of Medical Oncology, Chiba University Hospital, Chiba, Japan; 22 Department of Otorhinolaryngology, Head and Neck Surgery, Chiba University Hospital, Chiba, Japan; 23 Department of Head and Neck Surgery, Tokyo Medical and Dental University Medical Hospital, Tokyo, Japan; 24 Medical Affairs, Ono Pharmaceutical Co., Ltd., Osaka, Japan; 25 Japan Medical and Development, Bristol-Myers Squibb K.K., Tokyo, Japan; 26 Department of Otolaryngology, Head and Neck Surgery, Faculty of Medicine and Graduate School of Medicine, Hokkaido University, Kita15 Nishi7, Kita-Ku, Sapporo, Hokkaido 060-8638 Japan

**Keywords:** Nivolumab, Recurrent or metastatic head and neck cancer, Immune microenvironment, Cetuximab, Neck dissection

## Abstract

**Background:**

To examine the effect of prior use of cetuximab and neck dissection on the effectiveness of nivolumab, we conducted a large-scale subgroup analysis in Japanese patients with recurrent/metastatic head and neck cancer.

**Methods:**

Data on the effectiveness of nivolumab were extracted from patient medical records. All patients were analyzed for effectiveness by prior cetuximab use. In the analyses for prior neck dissection, only patients with locally advanced disease were included.

**Results:**

Of 256 patients analyzed, 155 had received prior cetuximab. Nineteen of 50 patients with local recurrence underwent neck dissection. The objective response rate was 14.7 vs 17.2% (*p* = 0.6116), median progression-free survival was 2.0 vs 3.1 months (*p* = 0.0261), and median overall survival was 8.4 vs 12 months (*p* = 0.0548) with vs without prior cetuximab use, respectively. The objective response rate was 23.1 vs 25.9% (*p* = 0.8455), median progression-free survival was 1.8 vs 3.0 months (*p* = 0.6650), and median overall survival was 9.1 vs 9.9 months (*p* = 0.5289) with vs without neck dissection, respectively.

**Conclusions:**

These findings support the use of nivolumab for patients with recurrent/metastatic head and neck cancer regardless of prior cetuximab use or neck dissection history.

**Trial registration number:**

UMIN-CTR (UMIN000032600), Clinicaltrials.gov (NCT03569436)

**Supplementary Information:**

The online version contains supplementary material available at 10.1007/s10147-021-01900-4.

## Introduction

Head and neck cancers (HNCs) are heterogeneous in nature and in 2018, affected over 887,000 patients globally [1]. In Japan, over 27,000 patients were diagnosed with HNC in 2017 [2].

Nivolumab, a fully human immunoglobulin G4 monoclonal antibody targeting programmed death-1 (PD-1), was approved in March 2017 in Japan for the treatment of patients with recurrent or metastatic (R/M) HNC who were treated by platinum-based chemotherapy [3] based on the survival benefits and manageable safety profile demonstrated in a global Phase III clinical trial (CheckMate 141) [4]. Currently, nivolumab has become the standard treatment for patients who have experienced recurrence within 6 months of treatment with platinum-based chemotherapy, and real-world outcomes in patients receiving nivolumab have been reported [5–7]. We also reported the largest real-world evidence for the effectiveness and safety of nivolumab in a retrospective chart review study [8]. Our results for effectiveness, including best overall response (BOR), progression-free survival (PFS), overall survival (OS), and safety, were consistent with data from CheckMate 141 [4, 8].

Recently, the relationship between immune microenvironment and immunotherapy in patients with HNC has been under focus. Cetuximab, a monoclonal antibody against the epidermal growth factor receptor (EGFR), is often used as the first line of treatment for patients with R/M HNC as a part of the EXTREME regimen [9, 10]. Some reports have suggested that cetuximab may modulate the tumor microenvironment. Among patients with HNC receiving cetuximab monotherapy, immunosuppressive regulatory T cells were increased in those with a poor clinical outcome [11], while monocytic myeloid-derived suppressor cells (MDSCs) were significantly increased in nonresponders [12]. An ad hoc analysis of CheckMate 141 demonstrated that nivolumab had better efficacy than the investigator’s choice (IC) of chemotherapy in R/M HNC regardless of prior cetuximab use [13]. Of note, only 27 Japanese patients participated in this study; therefore, the effects of prior cetuximab use in Japanese patients were not investigated. Several studies have investigated the association between the outcome of nivolumab treatment and prior cetuximab use as one of the possible prognostic factors in Japanese patients with R/M HNC [6, 14–16]. However, the sample sizes of these studies were small.

Neck dissection is performed to remove metastatic lymph nodes and/or to prevent cancer recurrence in the neck. Preclinical data suggest that neck dissection may affect the efficacy of subsequent immunotherapy as a lymph node represents a pivotal meeting point of immune cells where adaptive immunity is induced [17, 18]. In mouse models, lymph node dissection reduced the effect of immunotherapy involving PD-1 and programmed death-ligand 1 (PD-L1) inhibitory antibodies [17]. However, the effect of neck dissection on immunotherapy in humans is still unclear due to a lack of analysis in clinical or observational studies.

Here, we analyzed the effect of prior use of cetuximab and neck dissection on the effectiveness of nivolumab in Japanese patients with R/M HNC in a real-world clinical setting by using a larger sample size compared with previous reports [6, 14–16].

## Patients and methods

### Patients

All patients with R/M (distant sites) HNC who had been treated with nivolumab for the first time between July 1, 2017, and December 31, 2017, were included. Patients who had previously participated in a clinical trial involving antineoplastic therapy were excluded. A total of 256 patients were analyzed in this study.

### Study design

This was a multicenter, noninterventional, retrospective study conducted at 23 centers in Japan. The full details of the study design have been published previously [8]. The study protocol was reviewed and approved by the Institutional Review Board/Independent Ethics Committee at each study site, and the study was conducted according to the ethical principles of the Declaration of Helsinki and the local regulations in Japan. Although informed consent was not obtained, patients were given the opportunity to decline permission for use of their clinical records for research (opt-out consent provision).

### Outcomes and assessments

The primary objectives were to determine the overall effectiveness, including BOR, PFS, and OS, and to evaluate immune-related adverse events in real-world clinical practice. The objective of this subgroup analysis was to evaluate the effect of prior use of cetuximab and neck dissection on the effectiveness of nivolumab by assessing BOR, PFS, and OS.

Data from baseline until the most recent patient visit were collected from patients’ medical charts using an electronic case report form. The baseline was defined as the visit before the start of nivolumab therapy. The data cutoff date was 1 year after the first treatment with nivolumab. Progression and response, primarily recorded by physicians, were measured according to investigator-assessed Response Evaluation Criteria in Solid Tumors (RECIST) version 1.1 criteria [19]. Evaluation time was not set because of the nature of this study.

### Statistical analyses

Analyses for effectiveness regarding prior cetuximab use were performed in all patients. In the analyses for effectiveness based on prior neck dissection, only patients with locally advanced disease were included to evaluate the influence of regional neck dissection on the effectiveness of nivolumab. Demographic and baseline characteristics and response data were summarized using descriptive statistics (number of patients, mean, and standard deviation) for continuous effectiveness variables and frequency and percentage for categorical variables. OS and PFS were estimated and plotted using the Kaplan–Meier method and expressed as the proportion of patients who survived to a specific point in time and median duration, with the corresponding two-sided 95% confidence intervals (CIs). Median OS and PFS were compared between the subgroups using the log-rank test. BOR was compared between the subgroups using the Wilcoxon rank-sum test, and objective response rate (ORR) and disease control rate (DCR) were compared between the subgroups using the Chi-square test. Statistical analyses were conducted using SAS version 9.4 (SAS Institute, Japan).

## Results

### Baseline demographics and disease characteristics

Overall patient baseline demographics and characteristics have been reported previously [8]. Briefly, of 256 patients, 202 (78.9%) were male, and the median age was 66 years (range: 20–84 years) (Table 1). Of all patients, 155 had received prior cetuximab treatment. Baseline disease characteristics were similar between patients with and without prior cetuximab exposure, with a few exceptions. Most patients with prior cetuximab exposure had received nivolumab treatment as a second-line (86 patients, 55.5%) or later-line (60 patients, 38.7%) therapy, while most patients without prior cetuximab exposure had received nivolumab treatment as first-line therapy (61 patients, 60.4%). Among the patients with prior cetuximab exposure, 21 (13.5%) had received cetuximab plus radiation (data not shown). Of the 50 patients with locally advanced disease, 31 (62%) had not undergone neck dissection (Table 1).

**Table 1:** Baseline demographics and disease characteristics

Characteristic	All patients	Prior cetuximab use		All patients with local recurrence	Prior neck dissection	
		With	Without		With	Without
All patients, *N* (%)	256 (100.0)	155 (100.0)	101 (100.0)	50 (100.0)	19 (100.0)	31 (100.0)
Sex						
Male, *n* (%)	202 (78.9)	129 (83.2)	73 (72.3)	35 (70.0)	15 (78.9)	20 (64.5)
Age, median (range), years	66 (20–84)	66 (20–84)	66 (24–80)	66 (29–78)	63 (34–72)	67 (29–78)
≥ 75 years, *n* (%)	24 (9.4)	13 (8.4)	11 (10.9)	5 (10.0)	0 (0.0)	5 (16.1)
ECOG PS, *n* (%)						
0	118 (46.1)	67 (43.2)	51 (50.5)	24 (48.0)	9 (47.4)	15 (48.4)
1	97 (37.9)	64 (41.3)	33 (32.7)	17 (34.0)	7 (36.8)	10 (32.3)
≥ 2^a^	31 (12.1)	20 (12.9)	11 (10.9)	6 (12)	2 (10.5)	4 (12.9)
Unknown	10 (3.9)	4 (2.6)	6 (5.9)	3 (6.0)	1 (5.3)	2 (6.5)
Nivolumab treatment line, *n* (%)						
1st line	70 (27.3)	9 (5.8)	61 (60.4)	15 (30.0)	6 (31.6)	9 (29.0)
2nd line	110 (43.0)	86 (55.5)	24 (23.8)	20 (40.0)	7 (36.8)	13 (41.9)
≥ 3rd line	76 (29.7)	60 (38.7)	16 (15.8)	15 (30.0)	6 (31.6)	9 (29.0)
Primary tumor site, *n* (%)						
Maxillary sinus^a^	14 (5.5)	10 (6.5)	4 (4.0)	5 (10.0)	1 (5.3)	4 (12.9)
Oral cavity	56 (21.9)	30 (19.4)	26 (25.7)	13 (26.0)	10 (52.6)	3 (9.7)
Salivary gland^a^	23 (9.0)	10 (6.5)	13 (12.9)	0 (0.0)	0 (0.0)	0 (0.0)
Larynx	21 (8.2)	15 (9.7)	6 (5.9)	4 (8.0)	1 (5.3)	3 (9.7)
Nasopharynx^a^	19 (7.4)	7 (4.5)	12 (11.9)	3 (6.0)	0 (0.0)	3 (9.7)
Oropharynx	40 (15.6)	26 (16.8)	14 (13.9)	11 (22.0)	4 (21.1)	7 (22.6)
Hypopharynx	64 (25.0)	48 (31.0)	16 (15.8)	9 (18.0)	3 (15.8)	6 (19.4)
Others	19 (7.4)	9 (5.8)	10 (9.9)	5 (10.0)	0 (0.0)	5 (16.1)

*ECOG PS* Eastern Cooperative Oncology Group performance status

^a^Not included in CheckMate 141 [4]

### Effectiveness outcomes based on prior cetuximab use

**Fig. 1:**
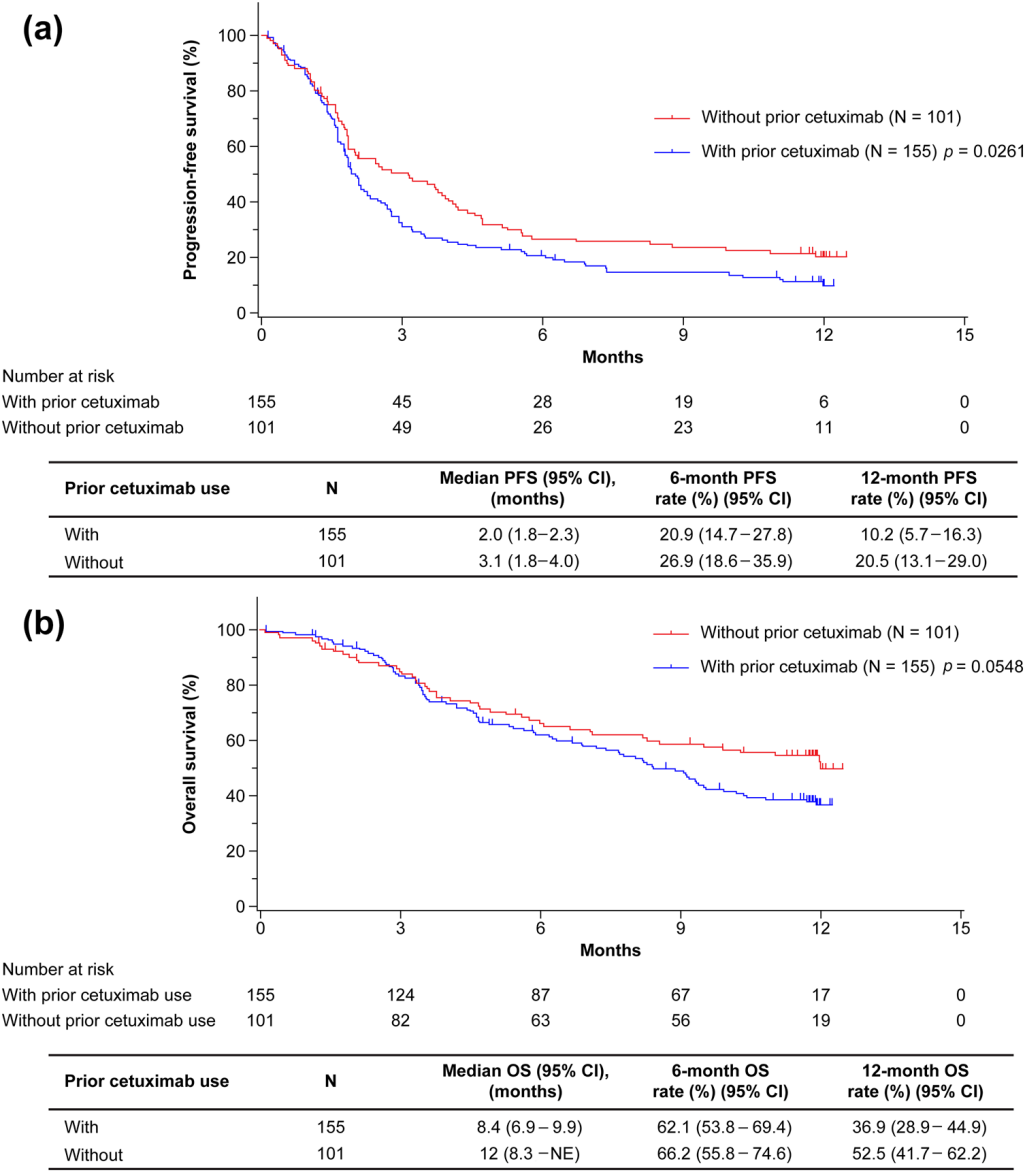
Overall effectiveness outcomes based on prior cetuximab use. **a** Progression-free survival in patients with and without prior cetuximab use. **b** Overall survival in patients with and without prior cetuximab use. *CI* confidence interval, *NE* not evaluable, *OS* overall survival, *PFS* progression-free survival

Among 256 patients, the ORRs in patients with prior cetuximab exposure and without prior cetuximab exposure were 14.7% (95% CI, 9.2–21.8) and 17.2% (95% CI, 10.0–26.8), respectively (*p* = 0.6116; Table 2). The median PFS was 2.0 months (95% CI, 1.8–2.3) for patients with prior cetuximab use and 3.1 months (95% CI, 1.8–4.0) for patients without prior cetuximab use (*p* = 0.0261), and the estimated 1-year PFS rates were 10.2% (95% CI, 5.7–16.3) and 20.5% (95% CI, 13.1–29.0) for patients with and without prior cetuximab use, respectively (Fig. 1a). The median OS was 8.4 months (95% CI, 6.9–9.9) and 12.0 months (95% CI, 8.3–not evaluable [NE]) for patients with and without prior cetuximab use, respectively (*p* = 0.0548), and the estimated 1-year OS rates were 36.9% (95% CI, 28.9–44.9) and 52.5% (95% CI, 41.7–62.2) for patients with and without prior cetuximab use, respectively (Fig. 1b).

**Table 2:** Objective response rate in patients with and without prior cetuximab use

Criteria	All patients	With prior cetuximab	Without prior cetuximab	*p* value
Number of patients, *N* (%)	256 (100.0)	155 (60.5)	101 (39.5)	–
BOR^a^, *n* (%)	223 (87.1)	136 (87.7)	87 (86.1)	0.0602
CR	3 (1.3)	1 (0.7)	2 (2.3)	
PR	32 (14.3)	19 (14.0)	13 (14.9)	
SD	61 (27.4)	31 (22.8)	30 (34.5)	
PD	127 (57.0)	85 (62.5)	42 (48.3)	
ORR^a^				
*n* (%)	35 (15.7)	20 (14.7)	15 (17.2)	0.6116
95% CI	(11.2–21.1)	(9.2–21.8)	(10.0–26.8)	
DCR^a^				
*n* (%)	96 (43.0)	51 (37.5)	45 (51.7)	0.0364
95% CI	(36.5–49.8)	(29.4–46.2)	(40.8–62.6)	

*BOR* best overall response, *CI* confidence interval, *CR* complete response, *DCR* disease control rate, *ORR* objective response rate, *PD* progressive disease, *PR* partial response, *RECIST* Response Evaluation Criteria in Solid Tumors, *SD* stable disease

^a^RECIST version 1.1

### Effectiveness outcomes based on prior neck dissection

**Fig. 2:**
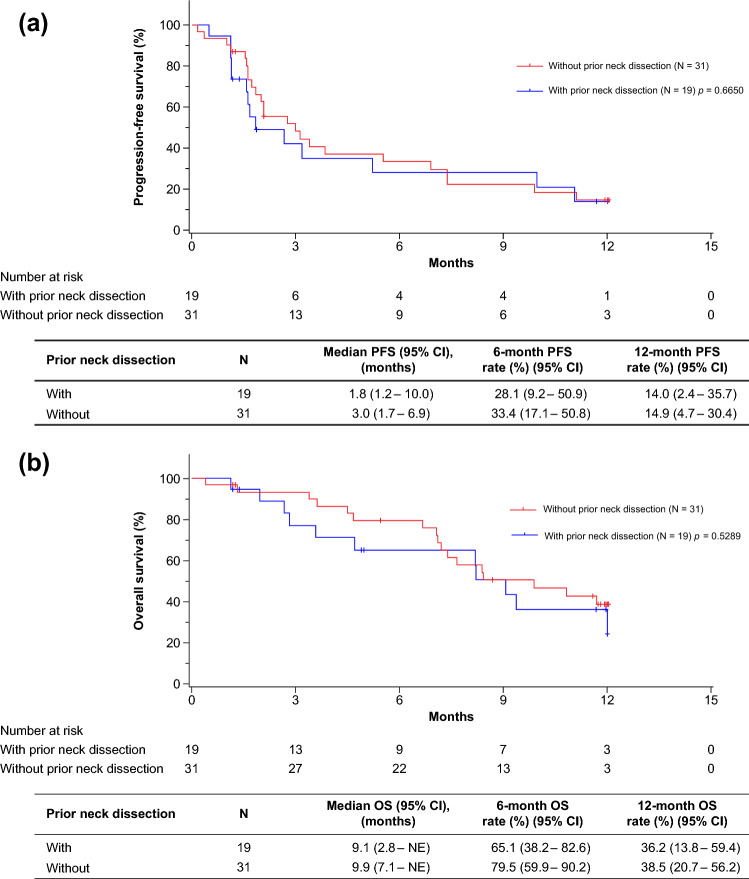
Overall effectiveness outcomes based on prior neck dissection. **a** Progression-free survival in patients with and without prior neck dissection. **b** Overall survival in patients with and without prior neck dissection. *CI* confidence interval, *NE* not evaluable, *OS* overall survival, *PFS* progression-free survival

Among 50 patients, the ORRs in patients with prior neck dissection and without prior neck dissection were 23.1% (95% CI, 5.0–53.8) and 25.9% (95% CI, 11.1–46.3), respectively (*p* = 0.8455; Table 3). The median PFS was 1.8 months (95% CI, 1.2–10.0) and 3.0 months (95% CI, 1.7–6.9) for patients with and without neck dissection, respectively (*p* = 0.6650), and the estimated 1-year PFS rates were 14.0% (95% CI, 2.4–35.7) and 14.9% (95% CI, 4.7–30.4) for patients with and without neck dissection, respectively (Fig. 2a). The median OS was 9.1 months (95% CI, 2.8–NE) and 9.9 months (95% CI, 7.1–NE) for patients with and without neck dissection, respectively (*p* = 0.5289), and the estimated 1-year OS rates were 36.2% (95% CI, 13.8–59.4) and 38.5% (95% CI, 20.7–56.2) for patients with and without neck dissection, respectively (Fig. 2b).

**Table 3:** Objective response rate in patients with and without prior neck dissection

Criteria	All patients	With prior neck dissection	Without prior neck dissection	*p*-value
Number of patients, *N* (%)	50 (100.0)	19 (38.0)	31 (62.0)	
BOR^a^, *n* (%)	40 (80.0)	13 (26.0)	27 (54.0)	0.7575
CR	0 (0.0)	0 (0.0)	0 (0.0)	
PR	10 (25.0)	3 (23.1)	7 (25.9)	
SD	7 (17.5)	2 (15.4)	5 (18.5)	
PD	23 (57.5)	8 (61.5)	15 (55.6)	
ORR^a^				
*n* (%)	10 (25.0)	3 (23.1)	7 (25.9)	0.8455
95% CI	(12.7–41.2)	(5.0–53.8)	(11.1–46.3)	
DCR^a^				
*n* (%)	17 (42.5)	5 (38.5)	12 (44.4)	0.7200
95% CI	(27.0–59.1)	(13.9–68.4)	(25.5–64.7)	

*BOR* best overall response, *CI* confidence interval, *CR* complete response, *DCR* disease control rate, *ORR* objective response rate, *PD* progressive disease, *PR* partial response, *RECIST* Response Evaluation Criteria in Solid Tumors, *SD* stable disease

^a^RECIST version 1.1

## Discussion

This is the first report of a large-scale retrospective study evaluating the effect of baseline characteristics such as prior use of cetuximab and neck dissection on nivolumab treatment in patients with R/M HNC in real-world clinical practice. Nivolumab showed effectiveness regardless of prior cetuximab use or neck dissection.

Cetuximab has been shown to promote differential innate and adaptive immune responses in patients with HNC treated with single-agent cetuximab [11, 12]. In these studies, cetuximab nonresponders showed an expansion of immunosuppressive regulatory T cells and monocytic MDSCs in the tumor microenvironment [11, 12]. Therefore, it was hypothesized that these immunosuppressive cell types would be present in patients who show disease progression after cetuximab therapy and may negatively impact a subsequent response to immunotherapy. However, the results of the current subgroup analysis were not completely in agreement with this hypothesis, and treatment effectiveness was similar irrespective of prior cetuximab use (Table 2 and Fig. 1). The results of the subgroup analysis in CheckMate 141 have been reported [13]. In patients with prior cetuximab use, the median OS (95% CI) was 7.1 months (4.9–8.7) with nivolumab vs 5.1 months (4.0–6.8) with IC. In patients without prior cetuximab use, the median OS (95% CI) was 8.2 months (5.4–9.9) with nivolumab and 4.9 months (3.1–6.5) with IC. Thus, the efficacy of nivolumab was demonstrated regardless of prior cetuximab use. In the current analysis, although the median OS for patients with prior cetuximab use was shorter than that for patients without prior cetuximab use (8.4 vs 12.0 months, *p* = 0.0548), there was no statistical significance between these two groups (Fig. 1b). Also, there was almost no difference in ORR between these two groups (14.7 vs 17.2%, respectively, *p* = 0.6116; Table 2). In contrast, the median PFS for patients with prior cetuximab use was significantly shorter than that for patients without prior cetuximab use (2.0 vs 3.1 months, respectively, *p* = 0.0261; Fig. 1); however, median PFS for patients with prior cetuximab use was similar to that for the overall population (2.1 months) reported previously [8]. Taken together, these results suggest that nivolumab is efficacious regardless of prior cetuximab use. Notably, there was a significant difference in the number of lines of prior systemic cancer therapy (Table 1), although ORR, median PFS, and OS values were similar irrespective of the nivolumab line of therapy (Online Resource 1). The different numbers of prior systemic therapy may also account for the absence of impact of prior cetuximab use on the effectiveness of nivolumab observed in this study.

In this analysis, no major difference in the effectiveness of nivolumab with respect to ORR, PFS, and OS was observed between patients with and without prior neck dissection (Table 3 and Fig. 2). It has been shown in mouse models that lymph node dissection reduces the effect of immunotherapy involving PD-1 and PD-L1 inhibitory antibodies [17]. In contrast, a recent study using mouse models has shown that lymph node dissection alongside established primary tumors did not affect response to immune checkpoint blockades for artificially recurrent tumors owing to the distribution of tumor-specific T cells from peripheral lymphatic organs [20]. To our knowledge, this is the first article reporting the effect of prior neck dissection on immunotherapy in humans. The results observed in our study are consistent with recent preclinical data [20]. A plausible reason why neck dissection did not affect the effectiveness of nivolumab in this study could be the contribution of other lymph nodes to lymphocyte migration toward the tumor site when regional lymph nodes were dissected in humans [21]. As the number of lymph nodes is considerably high in the head and neck region in humans compared with mice [22, 23], the effectiveness of nivolumab may not be impacted by neck dissection.

The inherent limitations of a retrospective, observational, real-world study design should be acknowledged. Owing to the retrospective, observational nature of the study, there was no control group. Our data were based on the assessments performed by individual physicians during their clinical practice, which could have resulted in some inconsistencies in the medical recording. We preferentially included study centers that had a greater number of patients with R/M HNC, with an intent to recruit an optimal number of patients. This may have unintentionally introduced a selection bias in the study population.

In conclusion, this large-scale subgroup analysis revealed that nivolumab was efficacious regardless of prior cetuximab use or neck dissection for patients with R/M HNC. This result supports the use of nivolumab for these patients regardless of prior cetuximab use or neck dissection history.

## Supplementary Information

Below is the link to the electronic supplementary material.

Supplementary file1 (PDF 343 kb)

## References

[1] Bray F, Ferlay J, Soerjomataram I (2018). Global cancer statistics 2018: GLOBOCAN estimates of incidence and mortality worldwide for 36 cancers in 185 countries. CA Cancer J Clin.

[2] Vital Statistics. National Cancer Center Japan. Available via https://ganjoho.jp/reg_stat/statistics/stat/summary.html. Accessed 9 Jun 2020

[3] ONO receives approval for OPDIVO^®^ (nivolumab) intravenous infusion for treatment of recurrent or metastatic head and neck cancer as a partial change in approved items of manufacturing and marketing approval in Japan. Ono Pharmaceutical Co., Ltd. Available via https://www.ono-pharma.com/sites/default/files/en/news/press/sm_cn170324.pdf. Accessed 20 Apr 2020

[4] Ferris RL, Blumenschein G, Fayette J (2016). Nivolumab for recurrent squamous-cell carcinoma of the head and neck. N Engl J Med.

[5] Okamoto I, Sato H, Kondo T (2019). Efficacy and safety of nivolumab in 100 patients with recurrent or metastatic head and neck cancer—a retrospective multicentre study. Acta Otolaryngol.

[6] Hori R, Shinohara S, Kojima T (2019). Real-world outcomes and prognostic factors in patients receiving nivolumab therapy for recurrent or metastatic head and neck carcinoma. Cancers (Basel).

[7] Sato Y, Fukuda N, Wang X (2020). Efficacy of nivolumab for head and neck cancer patients with primary sites and histological subtypes excluded from the CheckMate-141 trial. Cancer Manag Res.

[8] Hanai N, Shimizu Y, Kariya S (2021). Effectiveness and safety of nivolumab in patients with head and neck cancer in Japanese real-world clinical practice: a multicenter retrospective clinical study. Int J Clin Oncol.

[9] Graham J, Muhsin M, Kirkpatrick P (2004). Cetuximab. Nat Rev Drug Discov.

[10] Vermorken JB, Mesia R, Rivera F (2008). Platinum-based chemotherapy plus cetuximab in head and neck cancer. N Engl J Med.

[11] Jie HB, Schuler PJ, Lee SC (2015). CTLA-4(+) regulatory T cells increased in cetuximab-treated head and neck cancer patients suppress NK cell cytotoxicity and correlate with poor prognosis. Cancer Res.

[12] Li J, Srivastava RM, Ettyreddy A (2015). Cetuximab ameliorates suppressive phenotypes of myeloid antigen presenting cells in head and neck cancer patients. J Immunother Cancer.

[13] Ferris RL, Licitra L, Fayette J (2019). Nivolumab in patients with recurrent or metastatic squamous cell carcinoma of the head and neck: efficacy and safety in CheckMate 141 by prior cetuximab use. Clin Cancer Res.

[14] Nishikawa D, Suzuki H, Koide Y (2018). Prognostic markers in head and neck cancer patients treated with nivolumab. Cancers (Basel).

[15] Suzuki C, Kiyota N, Imamura Y (2020). Effect of tumor burden and growth rate on treatment outcomes of nivolumab in head and neck cancer. Int J Clin Oncol.

[16] Ueki Y, Takahashi T, Ota H (2020). Predicting the treatment outcome of nivolumab in recurrent or metastatic head and neck squamous cell carcinoma: prognostic value of combined performance status and modified Glasgow prognostic score. Eur Arch Otorhinolaryngol.

[17] Fransen MF, Schoonderwoerd M, Knopf P (2018). Tumor-draining lymph nodes are pivotal in PD-1/PD-L1 checkpoint therapy. JCI Insight.

[18] Gasteiger G, Ataide M, Kastenmüller W (2016). Lymph node—an organ for T-cell activation and pathogen defense. Immunol Rev.

[19] Eisenhauer EA, Therasse P, Bogaerts J (2009). New response evaluation criteria in solid tumours: revised RECIST guideline (version 1.1). Eur J Cancer.

[20] Zhao X, Kassaye B, Wangmo D (2020). Chemotherapy but not the tumor-draining lymph nodes determine the immunotherapy response in secondary tumors. iScience.

[21] Ellis RJ, Wernick G, Zabriskie JB (1975). Immunologic competence of regional lymph nodes in patients with breast cancer. Cancer.

[22] Koroulakis A, Agarwal M (Updated Nov 30, 2019). Anatomy, head and neck, lymph nodes. In: StatPearls. Treasure Island (FL): StatPearls Publishing; Jan 2020. Available via https://www.ncbi.nlm.nih.gov/books/NBK513317/. Accessed 9 Jun 2020 30020689

[23] Van den Broeck W, Derore A, Simoens P (2006). Anatomy and nomenclature of murine lymph nodes: descriptive study and nomenclatory standardization in BALB/cAnNCrl mice. J Immunol Methods.

