# Supplementary material for: Effectiveness of nivolumab affected by prior cetuximab use and neck dissection in Japanese patients with recurrent or metastatic head and neck cancer: results from a retrospective observational study in a real-world setting
Source: Int J Clin Oncol. 2021 Apr 8;26(6):1049–56. doi: 10.1007/s10147-021-01900-4 (PMC8134300; doi:10.1007/s10147-021-01900-4)
Supplement: Supplementary file 1 — Supplementary file1 (PDF 343 kb) [file 10147_2021_1900_MOESM1_ESM.pdf]

Supplementary Material

Online Resource 1a Overall survival in patients by treatment line for nivolumab

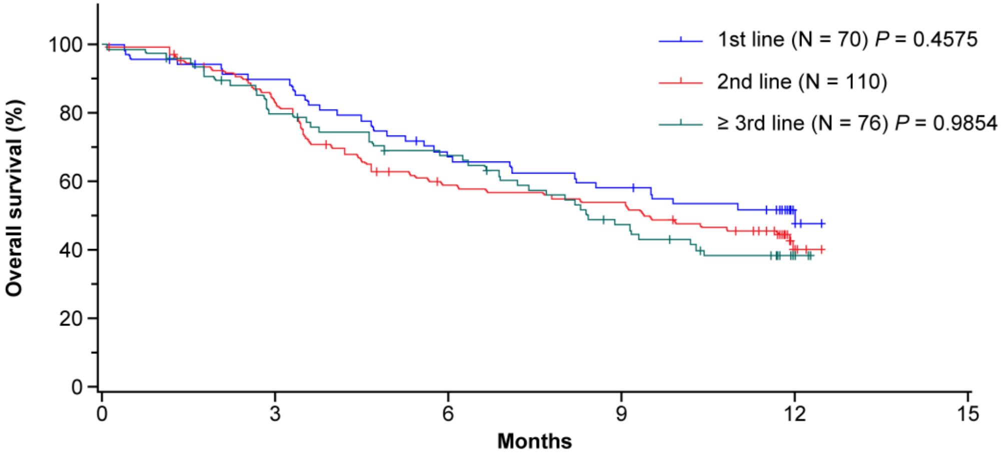

Number at risk						
1st line	70	60	44	38	13	0
2nd line	110	87	58	53	12	0
≥ 3rd line	76	59	48	32	11	0

**Prior lines of therapy**	**N**	**Median OS (95% CI), months**	**6-month OS rate (%) (95% CI)**	**12-month OS rate (%) (95% CI)**
1st line	70	12.0 (7.1, NE)	67.2 (54.6, 77.1)	51.8 (39.1, 63.0)
2nd line	110	9.4 (5.7, NE)	58.9 (48.8, 67.6)	40.2 (29.8, 50.4)
≥ 3rd line	76	8.4 (6.9, 10.4)	67.5 (55.5, 76.9)	38.3 (27.0, 49.5)

CI confidence interval, *NE* not evaluable, *OS* overall survival

Online Resource 1b Progression-free survival in patients by treatment line for nivolumab

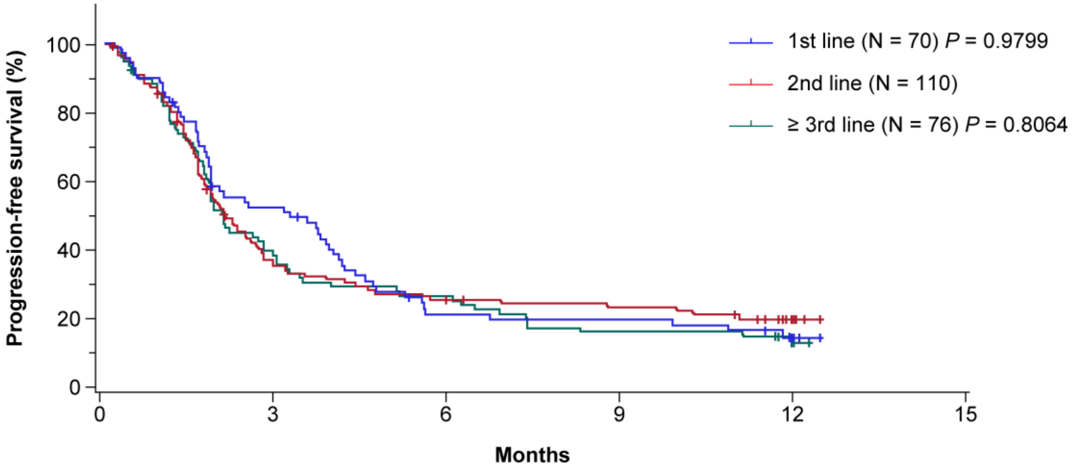

Number at risk						
1st line	70	34	12	11	4	0
2nd line	110	34	23	20	9	0
≥ 3rd line	76	26	19	11	4	0

Prior lines of therapy	N	Median PFS (95% CI), months	6-month PFS rate (%) (95% CI)	12-month PFS rate (%) (95% CI)
1st line	70	3.2 (1.8, 4.0)	20.1 (11.3, 30.6)	13.2 (6.2, 22.9)
2nd line	110	2.1 (1.7, 2.6)	24.2 (16.3, 32.8)	18.6 (11.6, 26.9)
≥ 3rd line	76	2.0 (1.7, 2.8)	25.4 (16.2, 35.6)	11.4 (5.4, 20.1)

CI confidence interval, *PD* progressive disease, *PFS* progression-free survival

Online Resource 1c Best objective response in patients by treatment line for nivolumab

Statistics			All	Nivolumab treatment line		
Category				1st line	2nd line	≥ 3rd line
Number of patients			256 (100.0%)	70 (27.3%)	110 (43.0%)	76 (29.7%)
The BOR assessed patient			223 (87.1%)	61 (23.8%)	98 (38.3%)	64 (25.0%)
BOR *1		CR	3 (1.3%)	0 (0.0%)	2 (2.0%)	1 (1.6%)
		PR	32 (14.3%)	9 (14.8%)	12 (12.2%)	11 (17.2%)
		SD	61 (27.4%)	21 (34.4%)	23 (23.5%)	17 (26.6%)
		PD	127 (57.0%)	31 (50.8%)	61 (62.2%)	35 (54.7%)
	ORR (%) *1	N (%)	35 (15.7%)	9 (14.8%)	14 (14.3%)	12 (18.8%)
		95% CI	(11.2 ,21.1)	(7.0 ,26.2)	(8.0 ,22.8)	(10.1 ,30.5)
	DCR (%) *1	N (%)	96 (43.0%)	30 (49.2%)	37 (37.8%)	29 (45.3%)
		95% CI	(36.5 ,49.8)	(36.1 ,62.3)	(28.2 ,48.1)	(32.8 ,58.3)

*1 RECIST ver.1.1

BOR best overall response, *CI* confidence interval, *CR* complete response, *DCR* disease control rate, *ORR* objective response rate, *PD* progressive disease, *PR* partial response, *RECIST* Response Evaluation Criteria in Solid Tumors, *SD* stable disease
